# Decoding the Elusive Redox Properties of [FeS] Clusters in [FeFe]-Hydrogenase on a Nanostructured Electrode

**DOI:** 10.1021/jacs.5c12671

**Published:** 2025-11-26

**Authors:** Yanxin Gao, Lei Wan, Serena DeBeer, Liyun Zhang, Olaf Rüdiger

**Affiliations:** † 28313 Max Planck Institute for Chemical Energy Conversion, Stiftstrasse 34-36, 45470 Mülheim an der Ruhr, Germany; ‡ State Key Laboratory of Medicinal Chemical Biology, College of Life Science, Nankai University, Tianjin 300350, China; § Nankai International Advanced Research Institute (Shenzhen Futian), Nankai University, Shenzhen, Guangdong 518045, China

## Abstract

Proton-coupled electron transfer (PCET) is essential for [FeFe]-hydrogenase to operate reversibly with high activity at a minimal overpotential. However, whether the reduction of the [4Fe4S] cluster is proton-coupled during the catalytic cycle remains under debate. Here, we employ indium tin oxide (ITO)-functionalized nanostructured electrodes to resolve the elusive non-turnover redox signals of all three [4Fe4S] clusters in *Desulfovibrio desulfuricans* [FeFe]-hydrogenase (*Dd*HydAB). Analysis of the redox properties of the [4Fe4S] clusters revealed a moderate pH dependence (∼30 mV/pH), suggesting that the reduction potential is modulated by second-coordination-sphere effects rather than direct protonation of the coordinating cysteine residues. Additionally, integration of the non-turnover signals yielded an accurate quantification of the enzyme’s concentration on the electrode surface. In combination with *in situ* maturation, this allowed us to quantitatively determine the turnover frequency of the enzyme on the electrode surface.

## Graphical abstract

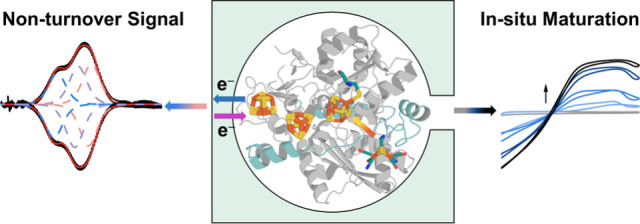

The [FeFe]-hydrogenases catalyze the reversible conversion of H_2_ with high efficiency and minimal overpotential requirements.^1,2^ The active site (H-cluster) of [FeFe]-hydrogenases consists of a canonical [4Fe4S] cluster ([4Fe4S]_H_) covalently linked via a cysteine thiolate to the coordinatively saturated proximal iron (Fe_p_) of the binuclear [2Fe] subcluster ([2Fe]_H_), leaving the distal iron (Fe_d_) with an open coordination site for binding substrates or inhibitors (Figure 1A). The two iron atoms in [2Fe]_H_ are bridged by a CO and a 2-azapropane-1,3-dithiolate (ADT) ligand, while the bridgehead nitrogen in the ADT plays a key role in facilitating the heterolytic cleavage of H_2_ at Fe_d_.^3^ Under catalytic conditions, the reduction of the most oxidized state, H_ox_ ([4Fe4S]_H_^2+^-(Fe­(II)­Fe­(I))), is proposed to yield two possible states, H_red_ ([4Fe4S]_H_^+^-Fe­(II)­Fe­(I)) or H_red_H^+^ ([4Fe4S]_H_^2+^-Fe­(I)­Fe­(I)), whose relative populations are pH dependent, with H_red_H^+^ arising from a protonation-coupled electronic rearrangement involving the ADT ligand (Figure 1B, Model 1).^4,5^ However, an alternative mechanism based on infrared (IR) spectroelectrochemical studies and density functional theory calculations proposed that [4Fe4S]_H_ acts as a protonation site during the catalytic cycle. This leads to the formation of additional [4Fe4S]_H_ protonated states, such as H_ox_H (H^+^[4Fe4S]_H_^2+^-Fe­(I)­Fe­(I)) and H_red_′ (H^+^[4Fe4S]_H_^+^-Fe­(I)­Fe­(I)) (Figure 1B, Model 2).^6–8^ These observations have sparked an ongoing debate regarding the catalytic cycle of [FeFe]-hydrogenases, specifically, whether proton-coupled electron transfer (PCET) occurs exclusively at the ADT ligand of the [2Fe]_H_ subcluster or also involves [4Fe4S]_H_.^8,9^

**Figure 1:**
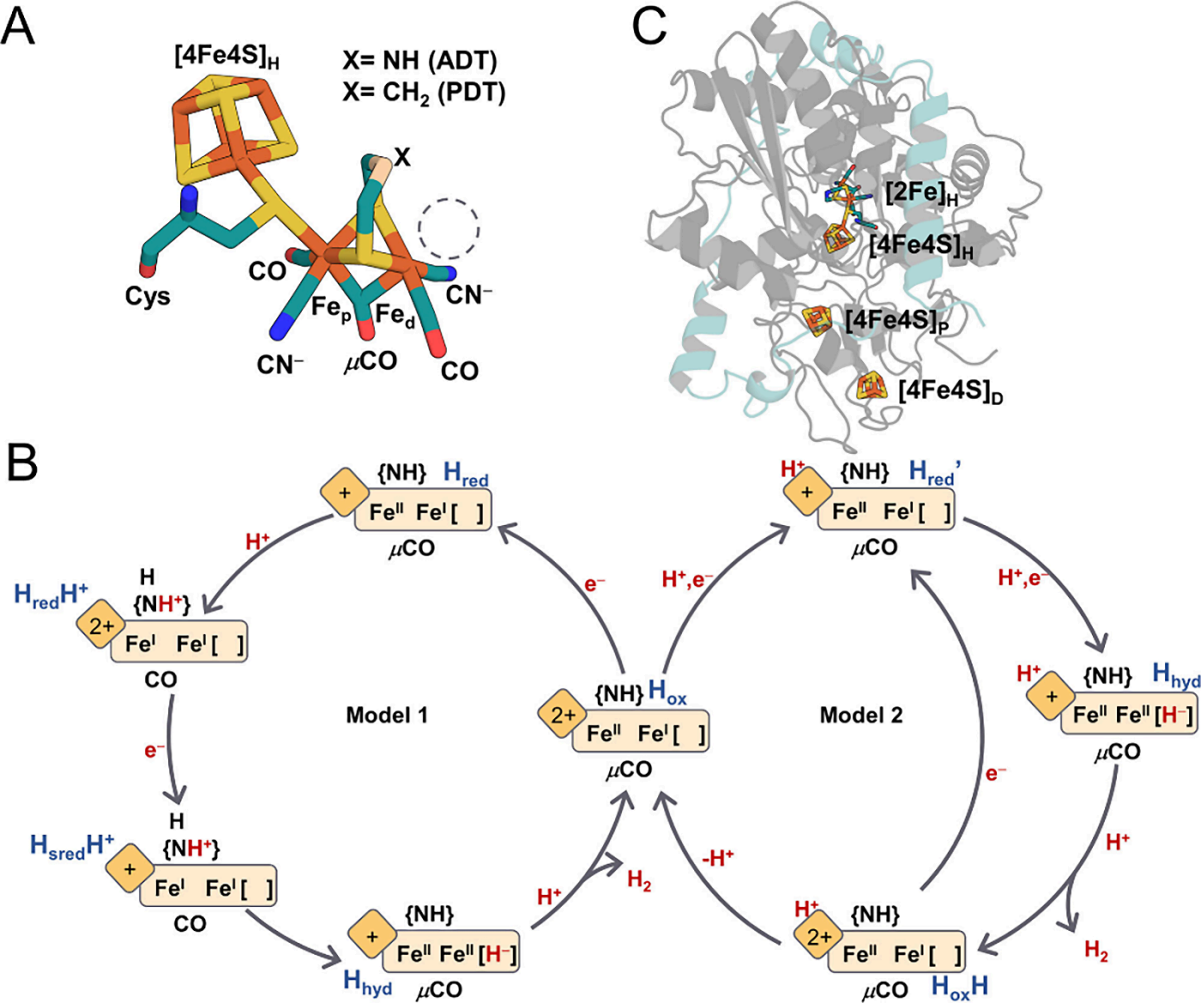
(A) Structural model of the H cluster. (B) Two simplified catalytic models of [FeFe]-hydrogenase operating in the direction of H_2_ evolution. [2Fe]_H_ and [4Fe4S]_H_ are represented as rectangles and squares, respectively. Bridging CO and ADT ligands are labeled as −CO and {NH}, respectively. Terminal CN^–^ and CO ligands are omitted for clarity. (C) Structure of *Dd*HydAB (PDB 1HFE). The HydA subunit is colored in gray, and the HydB subunit is colored in blue.

The heterodimeric [FeFe]-hydrogenase from *Desulfovibrio desulfuricans* (*Dd*HydAB) consists of two subunits with a small subunit (HydB) wrapped around the big subunit (HydA), which contains the H-cluster and two accessory [4Fe4S] clusters mediating long-range electron transfer (Figure 1C).^10,11^ Heterologous expression of *Dd*HydAB in *E. coli*, followed by *in vitro* maturation with [2Fe]_H_ subcluster synthetic analogue ([2Fe]^ADT/PDT^, PDT refers to propane-1,3-dithiolate), paves the way for detailed mechanism studies (Figure 1A). The nonprotonatable methylene bridgehead of [2Fe]^PDT^ does not allow for proton-coupled electron transfer to [2Fe]_H_, thereby restricting electron transfer to the [4Fe4S] clusters only. As a facile and robust approach to characterize the redox properties of metallocofactors (e.g., [FeS] clusters), protein film electrochemistry (PFE) has historically struggled to yield well-resolved non-turnover signals for hydrogenases with only two examples from CO-inhibited [NiFe]- and [NiFeSe]-hydrogenases reported.^12–15^ To this end, we have utilized a pyrolytic graphite electrode (PGE) functionalized with indium tin oxide (ITO) nanoparticles, a previously reported robust and versatile 3D nanostructured platform^16–18^ that enabled efficient attachment of *Dd*HydAB and detailed electrochemical investigation of the [4Fe4S] clusters’ redox behavior as well as the mechanism of maturation, which for *Dd*HydAB is notably slow and not yet fully understood.^19^

When the apo-*Dd*HydAB (lacking the [2Fe]_H_ subcluster) is immobilized on an ITO/PGE electrode (apo-*Dd*HydAB@ITO/PGE), the high protein coverage enables the cyclic voltammetry (CV) to clearly show two distinguishable and reversible redox features attributable to [4Fe4S] clusters, which is absent in the control without an enzyme (Figures 2A and S1). After baseline subtraction, these non-turnover signals displayed a similar peak-to-peak separation of 11 ± 5 mV and peak currents varied linearly with scan rate, indicating an efficient interfacial electron transfer between the ITO electrode and surface absorbed protein (Figures 2B and S2).^20,21^ The baseline-corrected redox profile was fitted with models assuming either two or three adsorbed one-electron redox species; however, only the latter model yields a satisfactory fit, consistent with three individually addressable [4Fe4S] clusters in apo-*Dd*HydAB (Figures 2B and S3). Previous studies have suggested that the redox anticooperativity between F cluster and H cluster in [FeFe]-hydrogenase shifts the H cluster cubane potential more negative than the proximal F cluster.^10,22^ However, the individual reduction potentials of the F clusters could not be directly determined. In this work, we experimentally determined three distinct redox transitions in the apo-protein at pH 7 with the reduction potentials of −472 ± 6 mV, −381 ± 7 mV, and −325 ± 10 mV vs SHE (Figure 2B). Based on correlations to previous studies (Figure S4A, for a comparison of the CVs with the reported potentials at pH 8.0)^10^ and the shifts in potential observed on a variant where one of the coordinating cysteines of the [4Fe4S]_D_ cluster was exchanged for an alanine (Figures S5 and S6), we assign these to the [4Fe4S]_H_, proximal [4Fe4S] cluster ([4Fe4S]_P_) and distal [4Fe4S] cluster ([4Fe4S]_D_), respectively (Figure 2C). The comparison of the catalytic wave and its first derivative from *Dd*HydAB^ADT^ with non-turnover signals revealed a clear overlap between catalytic wave and [4Fe-4S] clusters’ potentials, indicating that the protein environment fine-tunes the redox potential of these clusters relative to the pH-dependent 2H^+^/H_2_ potential to optimize catalysis in a wide pH range (Figure 2D and Figures S7 and S8).^23–27^

**Figure 2:**
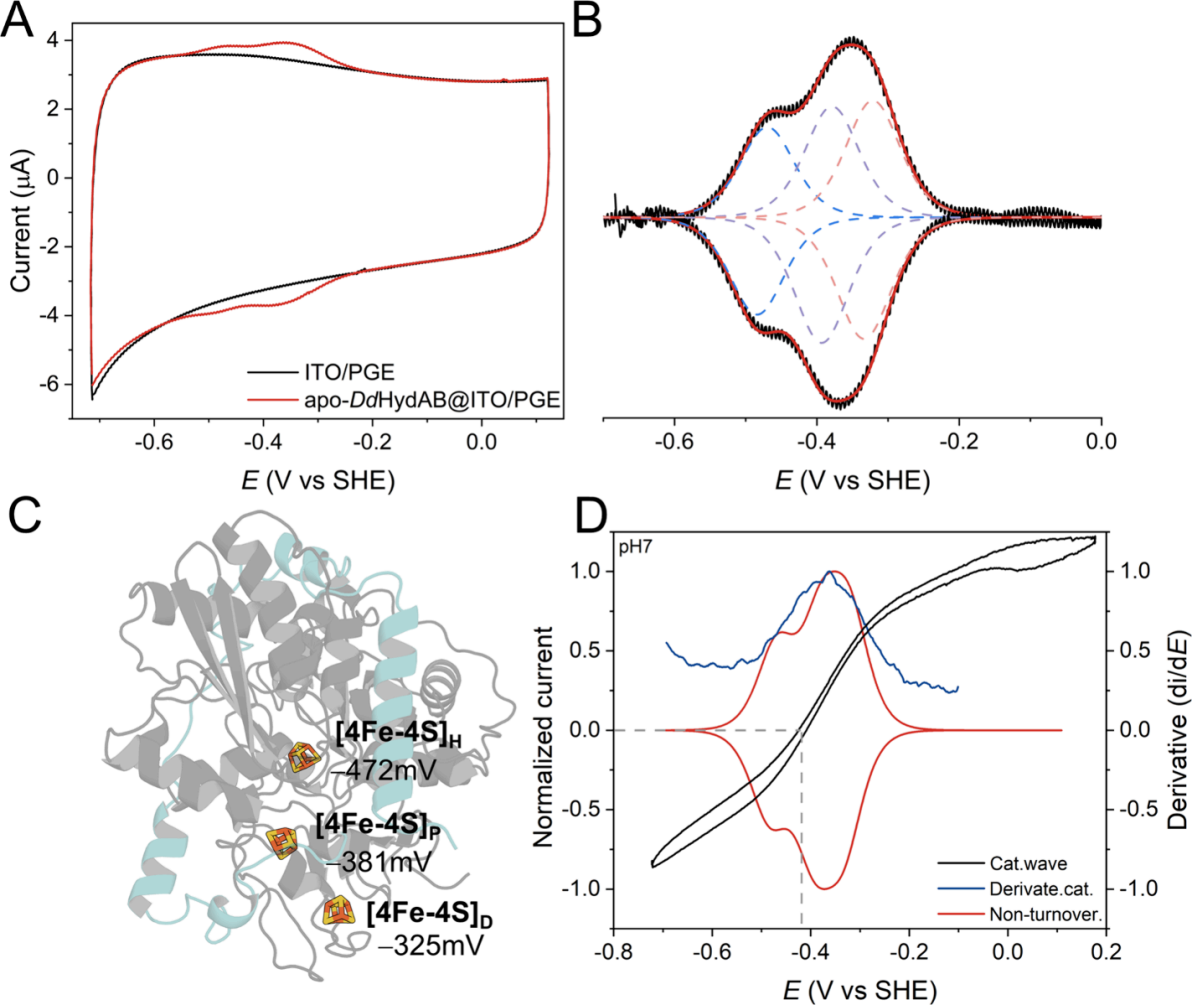
(A) Electrochemical response of an apo-*Dd*HydAB@ITO/PGE electrode (red) and an unmodified ITO/PGE electrode (black). (B) Background subtracted traces of the CV in (A), and the peak fitting result using Qsoas.^28^ (C) Reduction potential of three [4Fe4S] clusters at pH 7. (D) Comparison of normalized non-turnover signal (red), catalytic wave (black), and its first derivative (blue). The gray dashed vertical line represents the 2H^+^/H_2_ thermodynamic potential. The catalytic wave values are normalized to the H_2_ oxidation current overpotential of 300 mV. Conditions: scan rate at 50 mV s^–1^, 25 °C, pH 7; catalytic data was collected with PGE electrode under 100% H_2_ (1000 mL min^–1^, 1 bar) with electrode rotation rate of 2000 rpm.

A previous study by Senger et al. on *Chlamydomonas reinhardtii* (*Cr*HydA1) maturated with [2Fe]^PDT^ subcluster reported ∼55 mV/pH dependence in transition potential of the H_ox_H/H_red_′H and H_ox_/H_red_′ couples, suggesting the protonation of one cysteine ligand of [4Fe4S]_H_.^7^ However, a similar experiment conducted by Rodríguez-Maciá́ et al. did not find evidence for [4Fe4S]_H_ protonated species in both [FeFe]-hydrogenases [2Fe]^PDT^ variants from *Clostridium pasteurianum* (*Cp*I) and *Cr*HydA1, raising concerns that the additional species like H_ox_H might be experimental artifacts caused by reductants such as sodium dithionite (NaDT).^9,22,29^ Having resolved the potentials of the individual components in the electron transfer chain of *Dd*HydAB, we were able to revisit a controversial question: whether PCET also involves the [4Fe4S]_H_ cluster. However, a major challenge in characterizing hydrogenases by PFE studies is the unavoidable presence of a proton substrate in aqueous media. To suppress turnover and isolate non-turnover signals of [4Fe4S] clusters with the presence of the [2Fe]_H_ subcluster, we employed two different strategies: (1) CO inhibition of *Dd*HydAB^ADT^, forming H_ox_–CO and H_red_–CO states that block proton access; (2) a catalytically inactive variant, *Dd*HydAB^PDT^ (Figure S9).^10,30^ However, CO dissociates from Fe_d_ at potentials below −520 mV vs SHE, restoring proton reduction activity.^30,31^ This was evidenced by the appearance of small proton reduction currents, which blurred the non-turnover signals, in particular the one corresponding to [4Fe4S]_H_ (Figure S4). Nevertheless, it is clear that the most negative cluster signal in apo-*Dd*HydAB@ITO/PGE vanishes under CO, probably as a result of its shift to a more negative potential. Additionally the potential of the proximal and distal clusters shifts to more negative potential in agreement with what was observed in previous reports (Figure S4A).^10^ This observation supports our assignment of the most negative redox peak to the [4Fe4S]_H_ cluster in contrast to a study on *Cp*I hydrogenase that assigned the most negative potential to the most surface-exposed cluster.^32^ The electrochemical profile of *Dd*HydAB^PDT^@ITO/PGE closely resembled that of the apo-enzyme, consistent with the blocked electron transfer from [4Fe4S]_H_ to [2Fe]^PDT^ (Figure 3 and Figure S10).^33^ The non-turnover couples centered at −468 mV vs SHE (pH 7) can be attributed to the transition of H_ox_^pdt^/H_red_^pdt^. To directly investigate the redox behavior of [4Fe4S] clusters versus pH, the pH-dependent non-turnover CVs were performed for *Dd*HydAB^PDT^ and apo-*Dd*HydAB in the complete absence of NaDT to prevent the interference of sulfur dioxide.^9^ As shown in Figure 3, both constructs exhibited only a weak pH dependence with maximum slopes observed between pH 6 and 8 of 29 ± 1 mV/pH, 27 ± 2 mV/pH, and 26 ± 1 mV/pH for [4Fe4S]_H_, [4Fe4S]_P_, and [4Fe4S]_D_, respectively, in *Dd*HydAB^PDT^ and 32 ± 3 mV/pH, 31 ± 5 mV/pH, and 24 ± 6 mV/pH, respectively, in apo-*Dd*HydAB.

**Figure 3:**
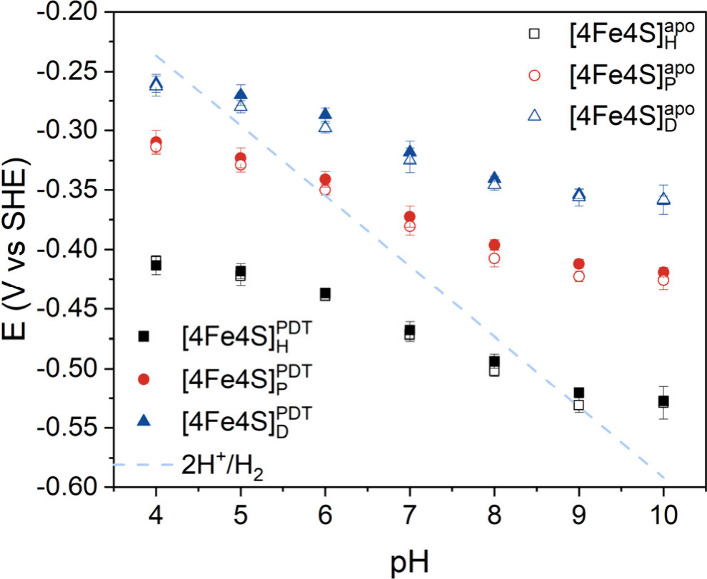
Reduction potential of three [4Fe4S] clusters in apo-*Dd*HydAB and *Dd*HydAB^PDT^ as a function of pH 4–10 with the standard 2H^+^/H_2_ reduction potential shown as a dashed blue line.

The observed weak pH dependence is incompatible with a direct protonation of the [4Fe4S]_H_ cluster or the effect from the ITO layer (Figure S11); instead, it likely arises from protonation events involving amino acid residues in the secondary coordination sphere (SCS) of the clusters that modulate the reduction potential through electrostatic interactions or hydrogen bonding.^27,34–37^

A small pH dependence for the potential of the three [4Fe4S] clusters has already been estimated from catalytic electrochemical methods by Leger and coworkers,^25,38^ in agreement with what we determined here. The profiles for all three [4Fe4S] clusters are quite similar, with the [4Fe4S]_H_ cluster having the most pronounced pH dependence and the distal cluster having a shallower profile (Figure S12). It is possible to roughly fit the data to a PCET model with two apparent p*K* values of 6 and 8 (approx.). This parallel behavior for all three clusters, with similar p*K* values, suggests a common mechanism regulating the redox potential with the pH for all three [4Fe4S] clusters. We are currently pursuing further studies to try to identify the amino acid residues responsible for this modulation of the redox potential of the FeS clusters. Such redox tuning is likely critical for [FeFe]-hydrogenase operations close to the thermodynamic 2H^+^/H_2_ potential, ensuring efficient catalysis at minimal overpotentials, as it can be seen in Figure S7, where the mismatch of potentials at pH 10 results in the enzyme losing bidirectionality and requiring a minimal overpotential to start H_2_ oxidation.

Encouraged by the well-resolved redox properties of [4Fe4S] clusters, we next aimed to investigate how these features relate to the incorporation of the [2Fe]_H_ subcluster and evaluate the influence of the redox environment on enzyme activation. As shown in Figure 4A, the non-turnover CV (gray line) was first collected, followed by the injection of [2Fe]^ADT^ (final concentration 1 μM) after which successive scans showed a progressive current increase in both directions, indicating *in situ* formation of active hydrogenase. The maturation process was also tracked by chronoamperometry (CA) to extract the kinetic information and assess the potential dependence of cofactor incorporation (Figure 4B and Figures S13 and S14). The variation in current suggests approximately first-order kinetics of subcluster incorporation with a half-life of maturation as 194 ± 32 s, which is a 50-fold rate increase compared with the enzyme maturated in solution.^19,39^ The accelerated maturation of *Dd*HydAB on the electrode is probably assisted by the nanoconfinement environment provided by the ITO electrode,^40^ which enriches [2Fe]^ADT^ in the nanopores (Figures S15 and S16; see Supplementary Discussion). Based on the protein coverage estimated by the integration of non-turnover signal, the apparent turnover frequency (apparent TOF) calculated from the measured electrocatalytic current at pH 6 was 227 ± 48 s^–1^ for proton reduction (at *E* = −0.63 V) and 181 ± 10 s^–1^ for hydrogen oxidation (at *E* = −0.05 V), values comparable to the solution matured *Dd*HydAB^ADT^ (Figure S17). These TOF values largely underestimate the intrinsic activity since they assume a 100% maturation of the apo-enzyme and that the substrate diffusion into the pores is not limited, which is not the case (see SI TOF discussion).

**Figure 4:**
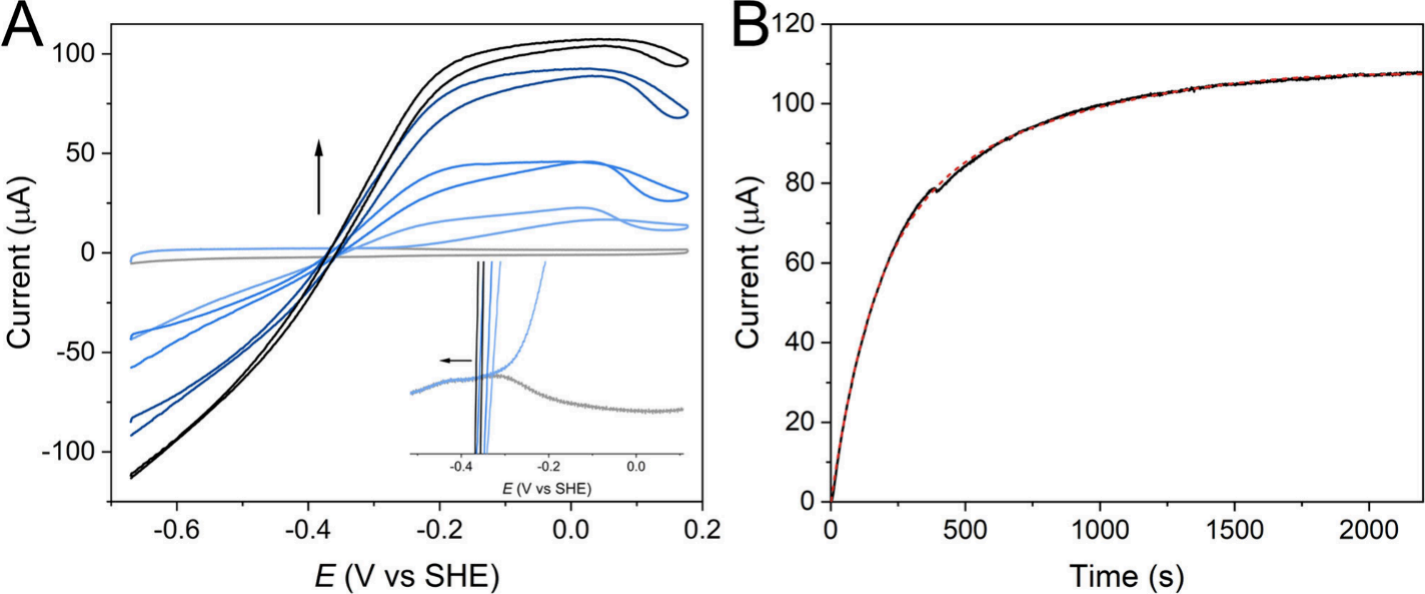
(A) Consecutive CVs (10 mV s^–1^) tracking the activation of apo-*Dd*HydAB adsorbed on an ITO/PGE electrode. Inset: enlarged view of the oxidative direction. (B) CA to monitor the activation process at 0 V vs SHE (black) with an exponential function fitted (red). [2Fe]^ADT^ (final concentration of 0.5 μM) was added at 0 s. Conditions: 2000 rpm rotating rate, 100% H_2_ (1000 mL min^–1^, 1 bar), 25 °C, pH 6.

In this work, we report a detailed electrochemical dissection of [4Fe4S] clusters in [FeFe]-hydrogenases using nanostructured ITO-functionalized electrodes. Using *Dd*HydAB as a model system, this represents, to the best of our knowledge, the first direct measure of resolved non-turnover signals in [FeFe]-hydrogenase and determination of reduction potentials in a F-cluster containing apo-protein. The pH-dependent experiments show a weak pH dependence (∼30 mV/pH) that may be influenced by residues from SCS, supporting the reduction of the [4Fe4S]_H_ cluster not being proton coupled.^22^ Since the potential dependence with pH does not significantly vary between the apo-*Dd*HydAB and *Dd*HydAB^PDT^ variants, it is likely that [2Fe]^PDT^ does not play a relevant role in the H^+^ transfer channel that modulates the potential of the FeS clusters. Further site-mutation experiments on SCS are needed to elucidate their precise roles in modulating redox properties. Additionally, the *in situ* activation also offers electrochemical insights into the maturation mechanism of *Dd*HydAB and determination of the TOF. Overall, this work offers a valuable framework for studying the redox behavior of metallocofactors and hydrogenase function, potentially guiding the design of bioinspired catalysts for efficient hydrogen conversion.

## Supplementary Material



## Data Availability

Data available at the Edmond Open Research Data Repository.^41^

## Notes

Materials and methods; supplementary PFE and FTIR figures and tables; supplementary discussions (PDF)
