# Supplementary material for: Decoding the Elusive Redox Properties of [FeS] Clusters in [FeFe]-Hydrogenase on a Nanostructured Electrode
Source: J Am Chem Soc. 2025 Nov 26;147(49):44661–6. doi: 10.1021/jacs.5c12671 (PMC12703649; doi:10.1021/jacs.5c12671)
Supplement: Supplementary file 1 [file ja5c12671_si_001.pdf]

Decoding the Elusive Redox Properties of [FeS] Clusters in [FeFe]-Hydrogenase on a Nanostructured Electrode

Yanxin Gao[1,2], Lei Wan[2,3], Serena DeBeer[1*], Liyun Zhang[2,3*], Olaf Ruediger[1*]

[1] Max Planck Institute for Chemical Energy Conversion, Stiftstrasse 34-36, 45470 Mülheim an der Ruhr, Germany.

[2] State Key Laboratory of Medicinal Chemical Biology, College of Life Science, Nankai University, Tianjin, 300350, China

[3] Nankai International Advanced Research Institute (Futian), Nankai University, Shenzhen, Guangdong, 518045, China

Material and methods

Production of apo-*Dd*HydAB and *Dd*HydAB$^{ADT/PDT}$.

*Dd*HydAB was produced recombinantly in *E. coli* BL21(DE3) ΔiscR and artificially maturated as described previously.[1] $(Et_4N)_2[Fe_2(PDT)(CN)_2(CO)_4]$ and $(Et4N)_2[Fe_2(ADT)(CN)_2(CO)_4]$ were prepared as described previously.[2,3] Site-directed mutagenesis at position C38A (numbering according to the expressed amino acid sequence) was performed by Quik Change using pACYCDuet-1(hyda/hydb) as the template plasmid[1] and the mutagenic primers list in Table S1. Protein concentrations were measured by UV-Vis absorbance spectroscopy using an extinction coefficient of 52.5 $mM^{-1}cm^{-1}$at 400 nm.[1] C38A-*Dd*HydAB concentrations were determined by the Lowry method using bovine serum albumin as a standard.[4] Protein purity was assessed by SDS-PAGE (Figure S5). Inductively coupled plasma mass spectrometry (ICP-MS) analysis confirmed the efficient maturation of *Dd*HydAB$^{ADT/PDT}$, revealing iron contents of 13.91±0.43 and 13.83±0.37 per protein for *Dd*HydAB^{ADT} and *Dd*HydAB^{PDT} , respectively.

The hydrogen oxidation activity of *Dd*HydAB^{ADT} was assessed by monitoring the reduction of 1 mM benzyl viologen at 600 nm (ε_{600} = 7.0 mM^{-1} cm^{-1}) under an H_2-saturated atmosphere.[1] Assays were conducted in 50 mM Tris-HCl buffer (pH 8) at 25 °C, with 0.1 nM *Dd*HydAB^{ADT}. The recorded hydrogen oxidation turnover rate was 71815 ± 6380 s^{-1}. Spectral measurements were performed in 1.5 mL UV-cuvettes using an Ocean Optics DH-mini UV-Vis-NIR light source and USB2000+XR1-ES detector, controlled by Spectra Suite software. Absorbance was monitored for up to 2 minutes, and activities were determined from the initial linear slope within the first 10–20 seconds of the reaction.

Preparation of ITO-PGE electrodes.

The preparation was carried out following a previously reported electrophoretic deposition (EPD) protocol.[5] Briefly, a 20 ml acetone suspension containing ITO (20 mg) (<50nm particle size, Sigma Aldrich) and iodine (5 mg) was sonicated for 45 minutes, then the counter and PGE electrode were held parallel in the suspension with a separation of 1–2 cm, and a voltage of 10 V was applied for 30s to 1 min.

Protein loading method.

The protein stock was first buffer exchangef to 10mM MES buffer pH 5.8, and diluted to 40μM. The bioelectrode was prepared by dropcasting 1μl 40μM apo-*Dd*HydAB or *Dd*HydAB$^{ADT/PDT}$ onto the electrodes, which were then incubated in the glovebox at room temperature for 2min and rinsed with water to remove any unbound protein. The resulting bioelectrodes were used immediately after preparation. Notably, the coverage of apo-*Dd*HydAB exhibited a pH-dependent behavior, with higher coverage at pH 5 compared to pH 9 (Figure S1). The higher affinity of *Dd*HydAB binding to ITO at lower pH is consistent with the isoelectric point of ITO films (~6), as at acidic pH the positively charged ITO surface favors electrostatic interactions with negatively charged residues surrounding the surface-exposed $[4Fe4S]_D$.[6,7]

Electrochemical measurement.

The cyclic voltammetry and chronoamperometry experiments were conducted in a two-compartment, three-electrode setup. The main compartment contains a working electrode and platinum (Pt) wire counter electrode, and was connected to the saturated calomel reference electrode (SCE, in 0.1M KCl) through a Luggin capillary. A VersaSTAT 4-400 potentiostat was used for all measurements, and the potentials were converted to the standard hydrogen electrode (SHE) using a conversion of +0.228 V from SCE. The temperature in the electrochemical cell was controlled by a water-jacketed system. All the experiments were performed in a buffer containing a mixture of 5 mM MES, HEPES, TAPS, CHES and sodium acetate as well as 0.1 M NaCl. The electrochemical data were processed in QSOAS[8], Origin and Python.

The non-turnover signals were analyzed by subtracting a baseline to remove the non-faradaic contributions. Since these signals are not very intense, the baseline correction process can introduce small errors in the peak intensities (but negligible error in the peak potential). To facilitate the fitting to an adsorbed species in QSOAS, coverage was allowed to float, resulting in slightly different peak areas for the three FeS clusters. To minimize this error, the average of the three peaks was used when calculating the enzyme coverage.

FTIR spectroscopy.

FTIR spectroscopy was performed on 10 μl samples placed between two CaF_2 windows (20 mm×4 mm, Korth Kristalle, Altenholz) separated by a 50mm Teflon spacer coated with vacuum grease and sealed in the FTIR cell with rubber o-rings. The FTIR samples were measured on a Bruker IFS 80v/S FTIR spectrometer equipped with a nitrogen-cooled Bruker mercury cadmium telluride (MCT) detector. Spectra were collected in the double-sided, forward backward mode, with a resolution of 2 cm^{-1}, an aperture setting of 1.5 mm, and a scan velocity of 20 Hz. Data were processed in QSOAS.[8]

EPR spectroscopy

cw X-band EPR samples (150 μL) were prepared in a N_2 filled anaerobic glovebox and transferred to 4 mm (o.d.) quartz EPR tubes, frozen in liquid nitrogen. The measurements were performed on a Bruker ELEXSYS E500 CW EPR spectrometer with an Oxford Instruments ESR900 helium flow cryostat connected to an ITC503 temperature controller. The parameters used were: microwave frequency=9.58 GHz; power=2mW; time constant=81.92 ms; conversion time=81.92 ms; and modulation frequency=100 kHz. All other parameters varied and are given in the figure legends. EPR spectral simulations were performed using Easyspin.[9]

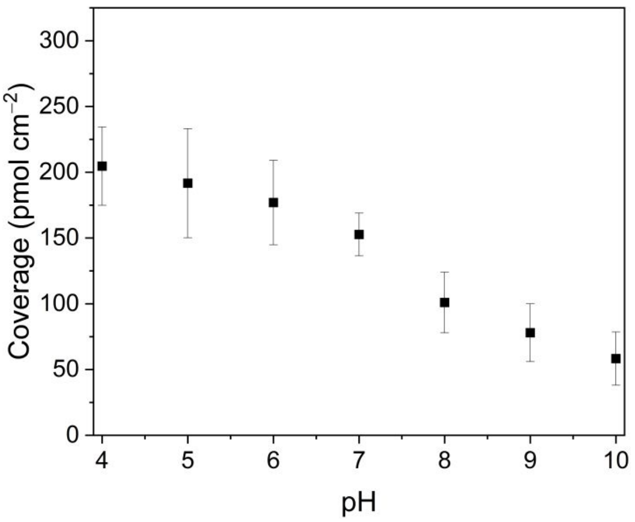

Figure S1. Calculated coverages of apo-*Dd*HydAB on ITO-PGE electrodes prepared at different pH values. The coverage is calculated as the average from the integration of the non-turnover signals for three independent electrodes, and the error bars indicate the standard deviation.

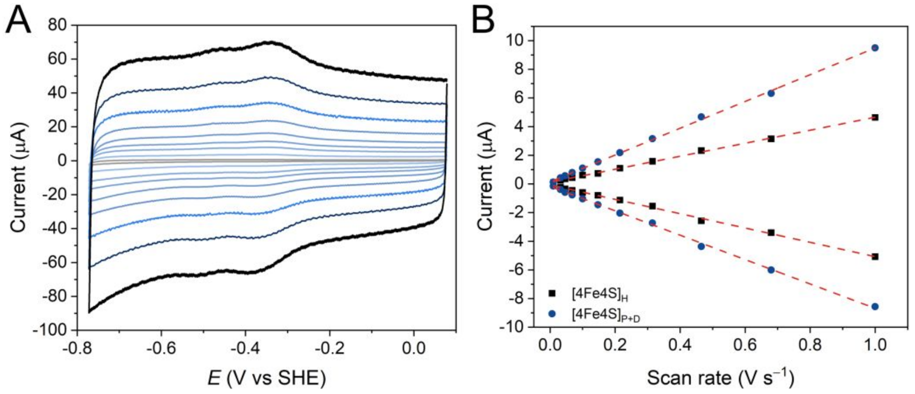

Figure S2. (A) Cyclic voltammograms of apo-*Dd*HydAB immobilized on ITO-functionalized pyrolytic graphite electrodes (ITO/PGE) recorded at scan rates ranging from 0.01 to 1 V s^{-1}. (B) Linear fits of cathodic and anodic peak currents versus scan rate after baseline correction, consistent with the behavior of absorbed redox species. Black squares correspond to the reversible redox peak assigned to the [4Fe4S]$_H$ cluster; blue circles correspond to the overlapping peaks of [4Fe4S]$_P$ and [4Fe4S]$_D$. Each data point represents the average of three independent electrodes. Measurements were performed at 25 °C in a mixed buffer (5 mM, pH 7.0) containing 0.1 M NaCl.

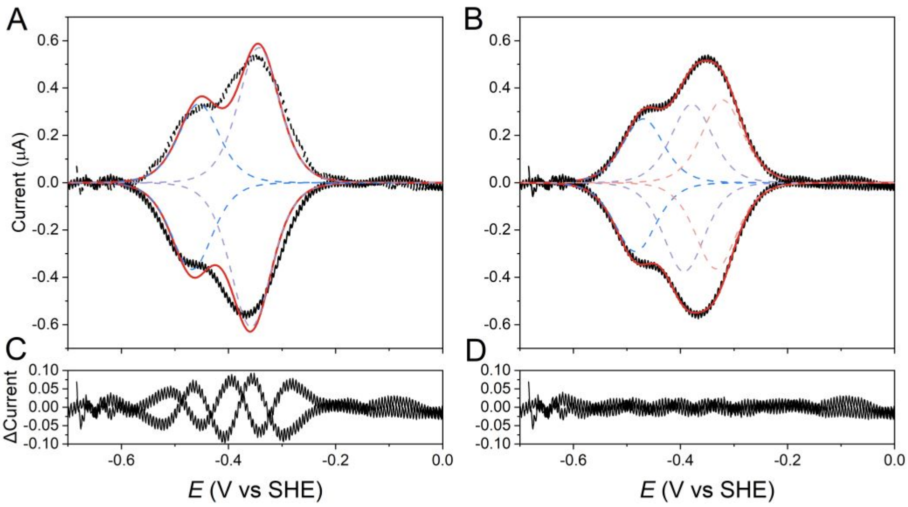

Figure S3. The non-turnover signal shown in Figure 2A was baseline-corrected and fitted in Qsoas[8] using models with either two or three adsorbed redox species. The corresponding results are shown in Figure S3A and Figure 3B, respectively. In both models, the number of electrons transferred (n) was fixed at 1, reflecting the intrinsic single-electron redox behavior of [4Fe4S] clusters in [FeFe]-hydrogenases. The two-species model (Figure S3A) failed to adequately reproduce the experimental data, yielding large residuals (Figure S3C), and the peak area of the positive potential peak is approximately 1.7 times larger, suggesting the overlapping of two one-electron centers with similar reduction potentials. In contrast, the three-species model (Figure S3B) yielded an excellent fit, with comparable peak areas across all redox events, consistent with the presence of three individually addressable [4Fe4S] clusters in apo-*Dd*HydAB.

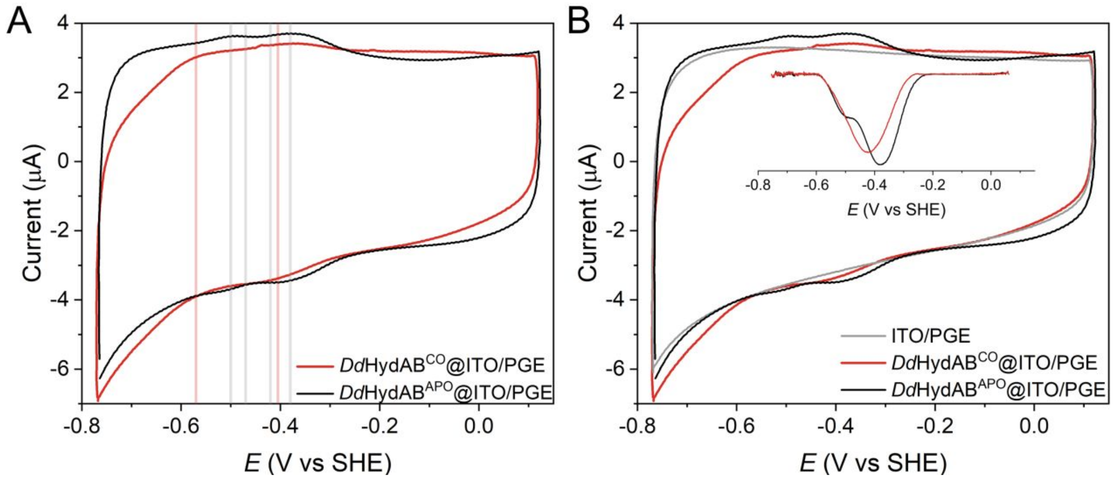

Figure S4. (A) Cyclic voltammetry showing the electrochemical response of ITO-PGE electrode loaded with *Dd*HydAB^{APO} (black), CO-inhibited *Dd*HydAB^{ADT} (red). The vertical lines represent the reported fitted redox potential of CO-inhibited *Dd*HydAB^{ADT} (red) and *Dd*HydAB^{ADT} respectively (black).[10] (B) Comparison of the *Dd*HydAB^{APO} (black), CO-inhibited *Dd*HydAB^{ADT} (red) in (A) with bare ITO-PGE electrode (black). The inset shows the corresponding baseline corrected cathodic traces. The ITO-PGE electrode was incubated with *Dd*HydAB^{ADT} (1 µl, 40 µM protein stock in 10 mM MES buffer, pH 5.8) for 2 min then rinsed with water to remove unbound protein. Conditions: 100% CO (1000 ml min^{-1}, 1 bar) purged into the cell headspace, 25 ºC, pH 8 and scan rate was 50 mV s^{-1}.

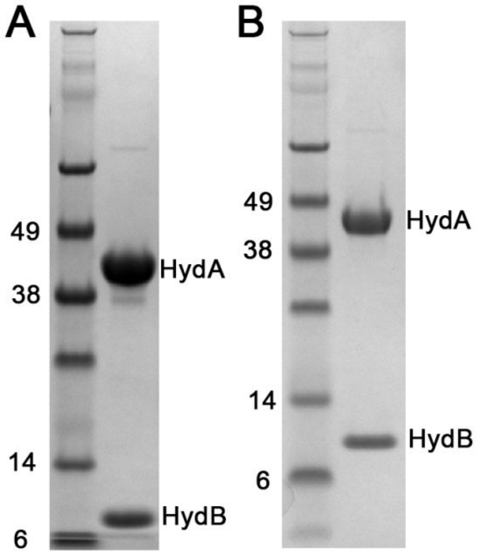

Figure S5. SDS-PAGE gel of the purified Samples of the purified wild-type apo-*Dd*HydAB (A), C38A apo-*Dd*HydAB (B) and the size standard (SeeBlue Plus2 Prestained Protein Standard, ThermoFisher), were loaded into the wells of a 4-12% Bis-Tris gel and electrophoresis was performed at 200 V, 60 mA, 1 h at room temperature in SDS-MES buffer.

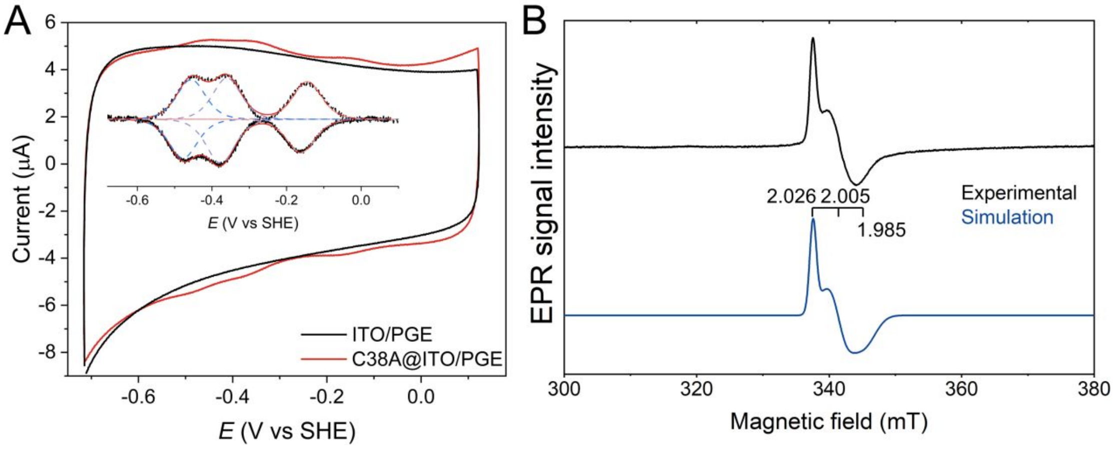

Figure S6. (A) CVs of the unmodified ITO-PGE electrode (black) and the ITO-PGE electrode loaded with C38A-*Dd*HydAB^{APO} (red). This variant was constructed by mutating the $[4Fe4S]_D$ coordinated cysteine (C38) to alanine (A38). This mutation has resulted in a clear positive shift of the reduction potential of $[4Fe4S]_D$ (from −325mV to −160mV), which supports the assignment of the most positive redox couple to the distal cluster. Inset shows the corresponding background subtraction and peak fitting of the CVs in (A). Conditions: scan rate at 50 mV s^{-1} recorded at 25 ºC, pH 7. (B) cw X-band EPR spectra of the 0.13mM C38A-*Dd*HydAB^{APO} sample, oxidized with 0.5 mM thionine, which shows the characteristic [3Fe4S] signal.

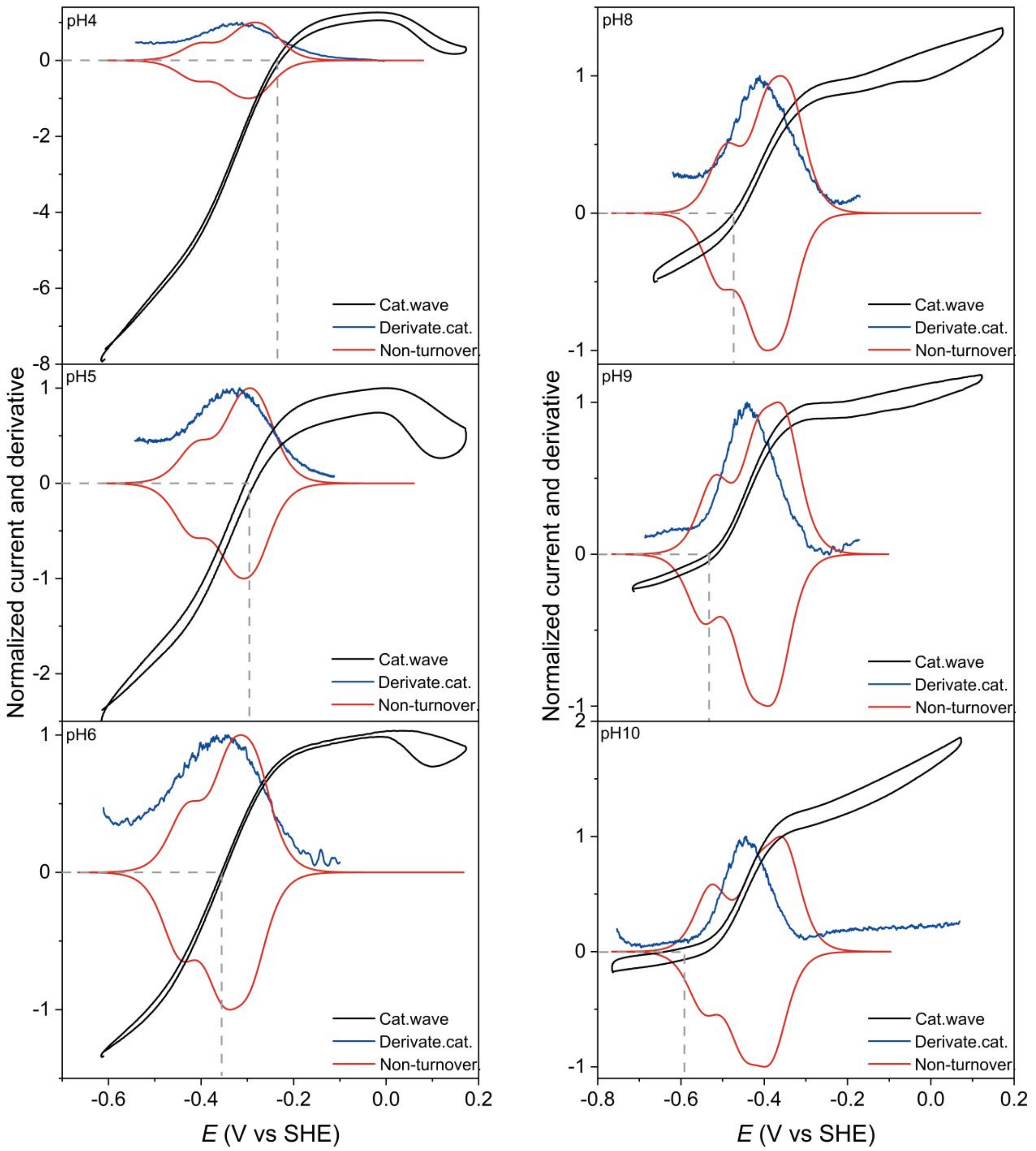

Figure S7. Comparison of normalized non-turnover signal (red), catalytic wave (black) and its first derivative(blue). The crossing point of the gray dashed lines represents the $2H^+/H_2$ thermodynamic potential. The catalytic waves of all pH values are normalized to the H_2 oxidation current overpotential of 300mV compared with $2H^+/H_2$ thermodynamic potential. Conditions: scan rate at 50 mV s^{-1}, 25 ºC, catalytic data was collected with PGE electrode under 100% H_2 (1000 ml min^{-1}, 1 bar) with electrode rotation rate of 2000 rpm.

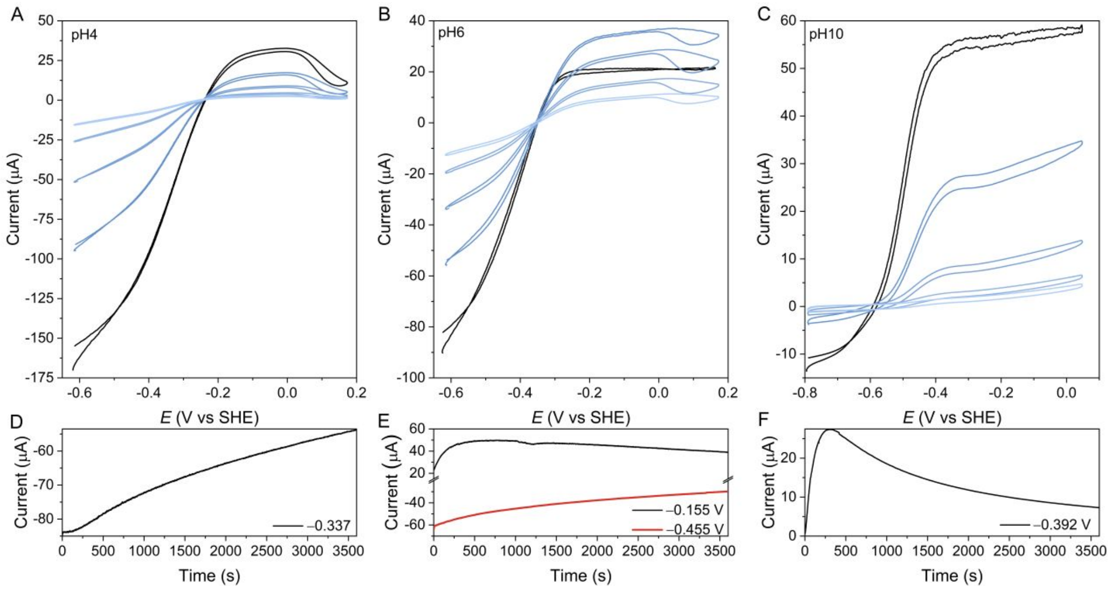

Figure S8. Electrochemical activity of *Dd*HydAB absorbed on PGE electrode at different pH values over prolonged periods. (A–C) Cyclic voltammetry (CV) of *Dd*HydAB/PGE at pH 4 (A), pH 6 (B), and pH 10 (C). (D) Chronoamperometry (CA) monitoring proton reduction by *Dd*HydAB/PGE at pH 4 with –100 mV applied potential relative to the $2H^+/H_2$ thermodynamic potential. (E) CA monitoring both proton reduction (red) and hydrogen oxidation (black) by *Dd*HydAB/PGE at –100 mV and +200 mV applied potentials, respectively, relative to the $2H^+/H_2$ thermodynamic potential. (F) CA monitoring hydrogen oxidation by *Dd*HydAB/PGE at +200 mV applied potential relative to the $2H^+/H_2$ thermodynamic potential. Experimental conditions: scan rate for CV: 50 mV s^{-1}; gas: 100% H_2 (1000 mL min^{-1}, 1 bar) for hydrogen oxidation in CA, CV and 100% N_2 (1000 mL min^{-1}, 1 bar) for proton reduction; electrode rotation rate: 2000 rpm; buffer: 5 mM mixture buffer with 0.1 M NaCl; temperature: 25 °C.

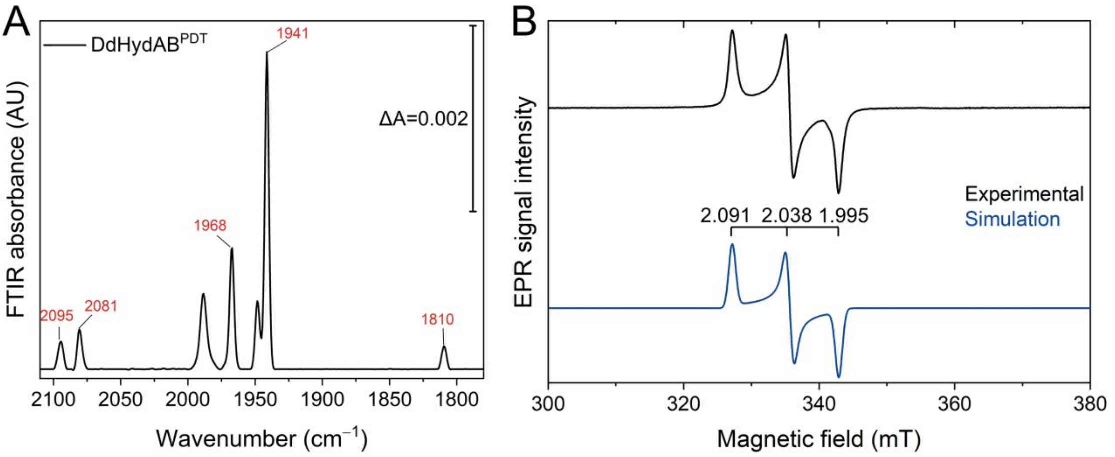

Figure S9. (A) FTIR spectra of as-prepared *Dd*HydAB^{PDT}. 80 μM in 25 mM Tris-HCl, 25 mM KCl, 5% glycerin, pH 8 under a 2 % H_2 atmosphere, and (B) cw X-band EPR spectra of the same sample, oxidized with 0.5 mM thionin, which shows the characteristic Hox signal.

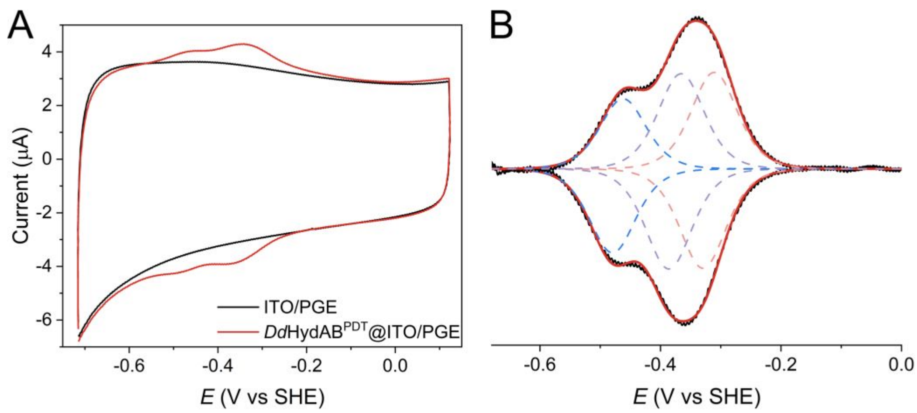

Figure S10. Electrochemical investigation of the redox behavior of [4Fe4S] clusters in *Dd*HydAB^{PDT}. (A) CVs of the unmodified ITO-PGE electrode (black) and the ITO-PGE electrode loaded with *Dd*HydAB^{PDT} (red). Conditions: scan rate at 50 mV s^{-1} recorded at 25 ºC, pH 7. (B) The corresponding background subtraction and peak fitting of the CVs in (A).

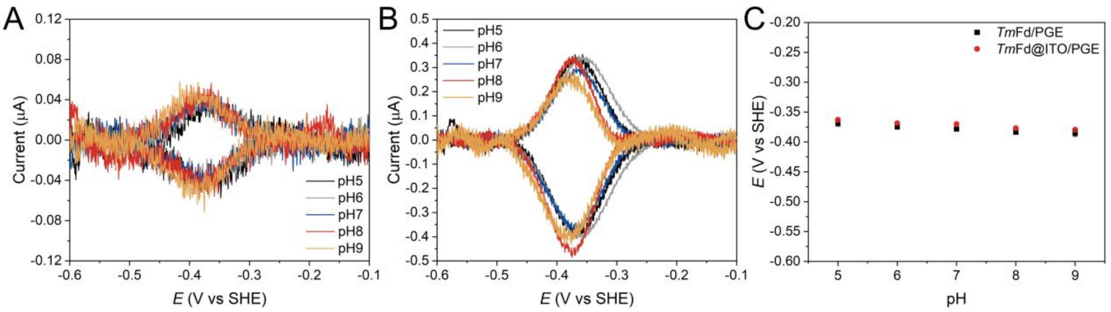

Figure S11. Electrochemical investigation of the redox behavior of *Tm*Fd. (A) CVs of PGE electrode loaded with *Tm*Fd at pH 5 to 9 after background subtraction (B) CVs of ITO-PGE electrode loaded with *Tm*Fd at pH 5 to 9 after background subtraction. (C) The reduction potential of *Tm*Fd as a function of pH 5-9. The PGE or ITO/PGE electrode was incubated with *Tm*Fd (1 μl, 40 μM protein stock in 10 mM MES buffer, pH 5.8) for 2 min then rinsed with water to remove unbound protein. Conditions: 25 ºC, 5mM mixture buffer charged with 0.1M NaCl, scan rate 100mV s^{-1}. The measured reduction potentials of *Tm*Fd at pH8 were −383 ± 2 mV for PGE and −377 ± 2 mV for ITO/PGE. Both values are close to the previous reported value of −388 mV at pH 8.[11] The observed pH-dependent reduction potential shifts were ~4 mV/pH for both electrodes, consistent with previous reports.[12] These results indicate that the presence of the ITO functional layer does not significantly alter the measured redox potentials or their pH dependence under our experimental conditions.

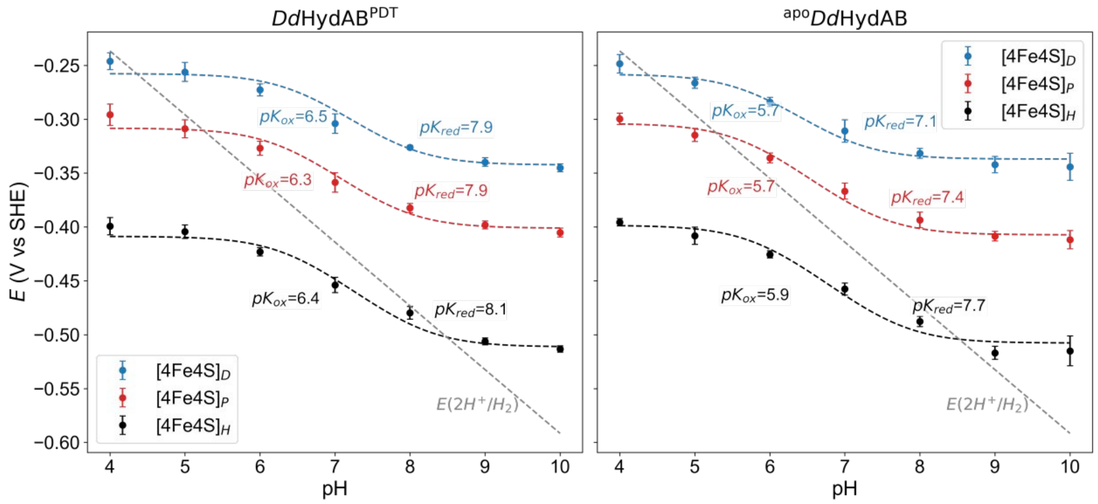

Figure S12. pH dependence of the potential of the [4Fe4S] clusters of the *Dd*HydAB^{PDT} and ^{apo}*Dd*HydAB variants. The dashed colored lines indicate a fitting to a PCET model corresponding to the following equation: $E\left([4Fe4S]_{ox}/[4Fe4S]_{red}\right) = E_{dep} - \frac{RT}{F}ln\left[\frac{1+10^{(pKox-pH)}}{1+10^{(pKred-pH)}}\right]$. The gray dashed line indicates the thermodynamic proton reduction potential, with a slope of 59 mV/pH. The obtained pK_{ox} and pK_{red} values are indicated in the figure.

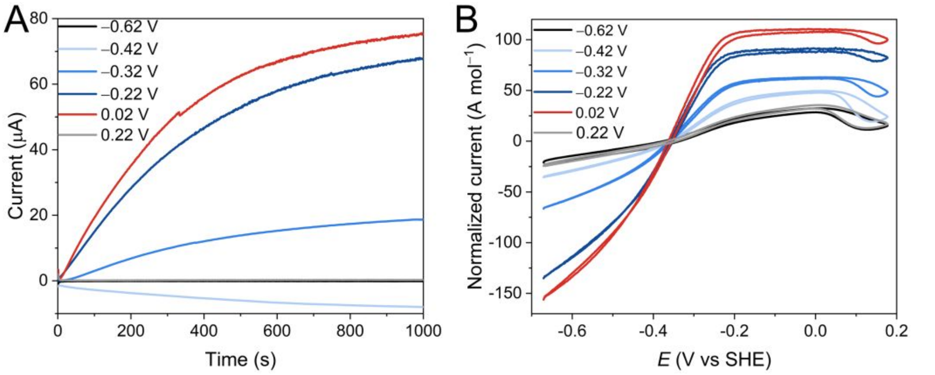

Figure S13. In vitro activation of apo-*Dd*HydAB observed by chronoamperometry under different potential and [2Fe]^{ADT} was injected to a final concentration of 0.5μM for initiating the activation (A). The activity of maturated *Dd*HydAB was compared by CV after 1000s activation. The current was normalized with protein coverage (B). Conditions: 100% H_2 (1000 ml min^{-1}, 1 bar), 2000rpm rotating rate, 10 mV s^{-1} scan rate.

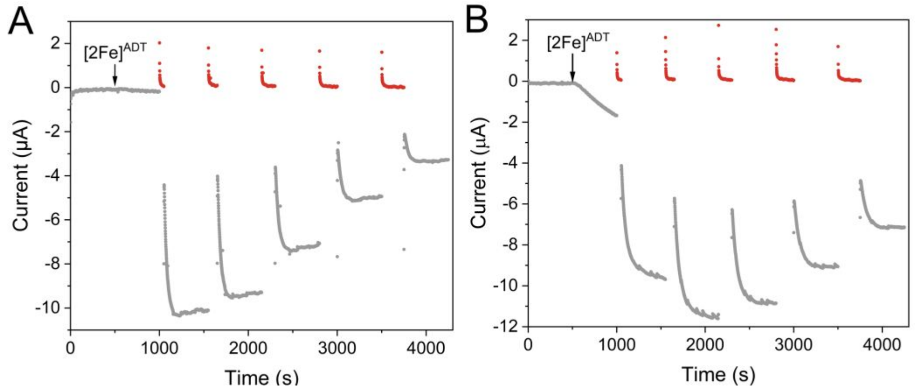

Figure S14. Voltage held at –0.554 V vs SHE (A) or –0.454 V vs SHE (B) initially for~1000 s (gray), during which $[2Fe]^{ADT}$ was injected to a final concentration of 0.5 μM at ~500 s. Potential stepped to 0 V (red) for five separate intervals and returned to the starting potential, –0.554 V (A) or –0.454 V (B), between each interval to monitor the H^+ reduction activity. The 100% N_2 was used to get rid of the H_2 uptake activity; electrode rotated at 2000 rpm. Other conditions: pH 6, 25 ºC. Taken the results from Figure S11 and S12 together, the activation process displays a strong potential dependence, with a threshold potential between –0.45V to –0.55V vs SHE, suggesting an oxidized $[4Fe4S]_H$ is the prerequisite for efficient cluster coupling (Figure S13 and S14), consistent with previous findings for *Cr*HydA1 and *Cp*I.[13,14]

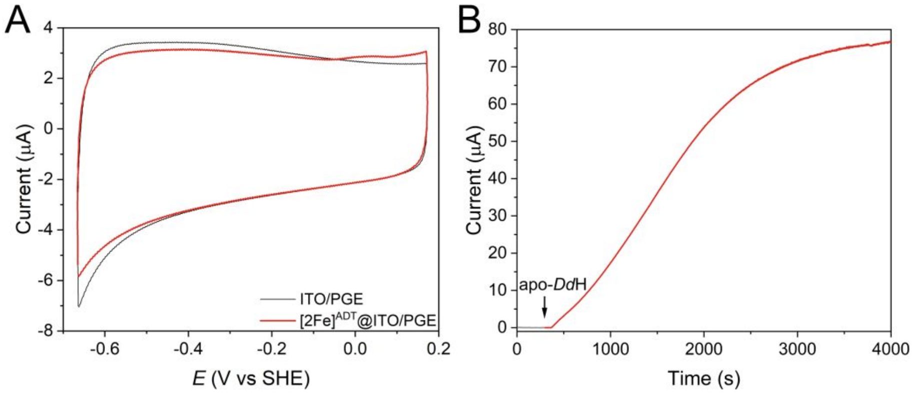

Figure S15. The ITO-PGE electrode was first loaded with 1.5 μl 2 mM $[2Fe]^{ADT}$ on the surface and incubated for 10 min, and rinsed with water thoroughly to remove any unbounded $[2Fe]^{ADT}$. (A) The CVs of the stationary unmodified ITO-PGE electrode (black) and the ITO-PGE electrode loaded with $[2Fe]^{ADT}$ (red). Conditions: 6ml buffer system, scan rate at 50 mV s^{-1} recorded at 25 ºC, pH 6 with 100% H_2 (1000 ml min^{-1}, 1bar). (B) The same electrode was held at 0.018 V vs SHE (grey), after 300 s, apo-*Dd*HydAB was injected into the electrolyte to give a final concentration of 1μM to initiate the activation (red). Conditions: identical to panel (A) except with electrode rotation at 2000 rpm.

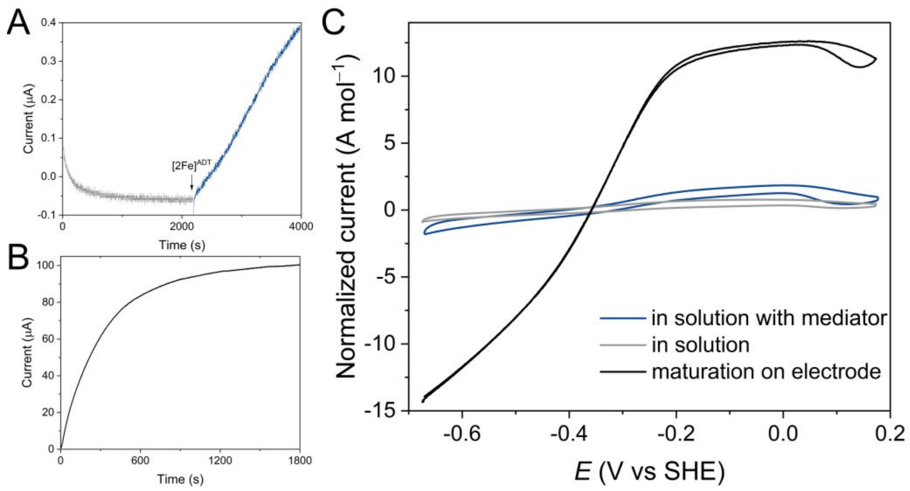

Figure S16. Activity comparison of apo-*Dd*HydAB maturated by different methods. (A) 6 ml of electrolyte containing 1.6 μM apo-protein, 16 μM methylene blue (E_m = +13 mV) and 16 μM indigo carmine (E_m = −125 mV) was first maintained at 0.018 V vs SHE for 2200s using a PGE working electrode to keep the dye oxidized. After that, the $[2Fe]^{ADT}$ was injected into the solution to a final concentration at 8μM to initiate the activation for 30min. Then the remaining ADT and mediator was removed using PD-10 column. The sample was collected by concentrator to 40μM and used for CV measurement in panel C (blue trace). (B) Electrochemical activation of apo-*Dd*HydAB on the ITO-PGE electrode, the potential was fixed at 0.018 V vs SHE and initiated by live injecting $[2Fe]^{ADT}$ to 0.5 μM at the beginning of the measurement. After 1800 s, the electrode was taken out and the buffer was exchange to fresh buffer without enzyme or ADT and the CV shown in panel C was measured (black line). (C) CV comparison of different activation method. For the gray trace in panel (C), 1.6 μM apo-protein was mixed with 8μM $[2Fe]^{ADT}$ in 2 ml buffer, and was incubated at room temperature for 1800 s, while stirring. Then remaining unreacted $[2Fe]^{ADT}$ was removed using PD-10 column, the sample was collected by concentrator to 40 μM. The currents in panel (C) were normalized with protein coverage. For all the maturation process, 100% H_2 (1000 ml min^{-1}, 1 bar) was maintained. Additional conditions for panel C: the ITO-PGE electrode was used as working electrode, pH6, 100% H_2 (1000 ml min^{-1}, 1 bar), 2000rpm rotating rate, 10 mV s^{-1} scan rate.

Supplementary discussion on the possible roles of ITO-electrodes in *Dd*HydAB maturation:

In this work, we observed that *Dd*HydAB matures significantly faster on the ITO-electrode compared to maturation in solution. While a detailed mechanistic understanding of how the ITO-electrode facilitates maturation remains elusive, several plausible explanations can be proposed.

One contributing factor is the ITO nanoparticles provided an effective interfacial electron transfer matrix for electron exchange between protein and electrode, as evidenced by the well-resolved non-turnover signal (Figure 2 and S2). Under these electrochemical conditions, controlled potential is applied, to ensure that the $[4Fe4S]_H$ is oxidized-ready for the $[2Fe]^{ADT}$. The other possible explanation could be that the nanostructured surface of ITO-electrodes provides a nanoconfined environment, which enables a "trapping" effect which keeps the *Dd*HydAB and the $[2Fe]^{ADT}$ inside the nanopores formed by the ITO nanoparticles. This local confinement likely increases the effective concentration of both components, enhances collision possibility, and limits diffusion losses into the bulk solution. While the concept of "nanoconfinement environment" is reminiscent of the "e-Leaf" , which emphasizes the limits of escaping of cofactor and subsequent intermediates during the multi-enzymatic cascade, here the focus is on facilitating protein-cofactor interactions during maturation.[15] Further support for this hypothesis is provided by the experiment shown in Figure S15, where the $[2Fe]^{ADT}$ was preloaded on the electrode and no electrocatalytic

$2H^+/H_2$ activity was observed. Even after thorough rinsing with deoxygenated water to remove unbound cofactor, the subsequent introduction of apo-*Dd*HydAB led to a notable increase in catalytic current. This indicates that the ITO electrode effectively retains $[2Fe]^{ADT}$ within its nanoporous structure and serves as a reservoir for cofactor delivery. The gradual rise in hydrogen oxidized current also suggests the apo-*Dd*HydAB or the activated holo-protein tend to diffuse from bulk solution into the nanopores. The prompt effect of ITO electrode on the *Dd*HydAB maturation is also confirmed by comparing catalytic activities of protein maturated by different conditions. Even when a solution-based system is maintained at oxidizing potential using redox mediators (Figure S16A), the measured electrocatalytic currents measured from these samples were lower than when the maturation was performed on the ITO electrode, which indicates a lower ratio of holoenzyme than that achieved on the ITO electrode (Figure S16C). Additionally, the higher proportion of active protein maturated in solution in the presence of oxidized mediator, further supports that an oxidized $[4Fe4S]_H$ is a prerequisite for efficient $[2Fe]^{ADT}$ binding and stable holoenzyme formation (Figure S16C).

Supplementary discussion on the measured apparent TOF values

The measured apparent TOF numbers are much lower than the reported rates for this enzyme measured in solution with redox mediators (63000±9000 s^{-1} for H_2 oxidation and 3700±400 s^{-1} for H_2 evolution).[1] Since the ICP-MS results (13.91±0.43 iron per protein for *Dd*HydAB^{ADT}) and solution assay for hydrogen oxidization (71815 ± 6380 s^{-1}) have confirmed a fully holo-*Dd*HydAB, several factors may contribute to this lower TOF, in addition to the partial maturation of the enzyme. One very clear is that there is mass transport limitation of substrate for both H_2 oxidation and evolution, since the current increases when increasing the rotation speed of the electrode (Figure S17). To estimate the diffusionless limit current, a Koutecky-Levich plot was used, where the intercept with the y-axis gives the inverse of the current in the absence of diffusion limitations (i_{lim}). In this case we obtain an i_{lim} of 1.7 mA for electrochemically maturated *Dd*HydAB, which for an enzyme coverage of 3.2 pmol, corresponds to an apparent diffusionless TOF of 2700 s^{-1}. A similar apparent diffusionless TOF of 2640 s^{-1} was obtained for the solution-maturated *Dd*HydAB, which had an enzyme coverage of 2.3 pmol and yielded an i_{lim} of 1.17 mA. This would account for the diffusion of H_2 and/or H^+ to or from the electrode surface, but not the diffusion through the pores. For three-dimensional electrodes with enzymes so active as hydrogenases, it has been reported that the H_2 is consumed on the film surface and it does not reach the inner layers of the pores, which would result in a lower TOF. This has been described for hydrogenases on carbon nanotubes[16], and also for hydrogenases in conductive polymer matrices[17-19].

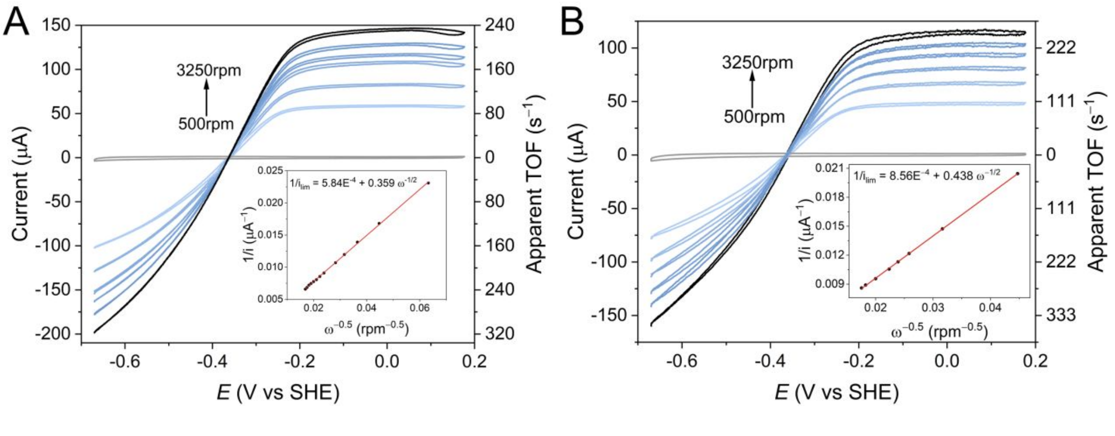

Figure S17. Cyclic voltammograms recorded at various rotation rates for electrochemically maturated *Dd*HydAB within the ITO-PGE electrode (A) or for an electrode where solution-maturated *Dd*HydAB was immobilized onto the ITO PGE electrode (B). To estimate the coverage of the solution-maturated *Dd*HydAB, 100% CO with flow rate at 200 ml min^{-1} was purged into the headspace to inhibit the catalytic current. With a known enzyme coverage, the

current can be converted to apparent TOF using the equation: $TOF = \frac{I}{nF\Gamma}$, where n corresponds to the number of electrons, which is 2 for H_2 oxidation/evolution; F is the faradic constant which is 96485 C mol^{-1}, Γ is the amount of the enzyme immobilized on the electrode surface (mol). Conditions: 100% H_2 (1000 ml min^{-1}, 1 bar), 25 ºC, pH 6 and 10 mV s^{-1} scan rate. The inset in (A) shows the Koutecky-Levich plot with the currents measured at -0.05 V and the linear fit to $1/i_{lim} = 5.85 \cdot 10^{-4} + 0.359 \cdot \omega^{-1/2}$. The inset in (B) shows the Koutecky-Levich plot with the currents measured at –0.05 V and the linear fit to $1/i_{lim} = 8.56 \cdot 10^{-4} + 0.438 \cdot \omega^{-1/2}$.

Table S1. Mutagenic primers used in this study

Name	Sequence 5' – 3'[a]	Nucleotides
C38A-F	GCATCGGC**gc**TGACACCTGCAGTCAATACT	30
C38A-R	CAGGTGTCA**gc**GCCGATGCATTTTGCTTCAT	31

[a] Bold, lower-case letters represent the mutated codons relative to the WT sequence.

Reference

(1) Birrell, J. A.; Wrede, K.; Pawlak, K.; Rodriguez-Maciá, P.; Rüdiger, O.; Reijerse, E. J.; Lubitz, W. Artificial Maturation of the Highly Active Heterodimeric [FeFe] Hydrogenase from Desulfovibrio Desulfuricans Atcc 7757. *Isr. J. Chem.* **2016**, *56* (9-10), 852-863.

(2) Li, H.; Rauchfuss, T. B. Iron Carbonyl Sulfides, Formaldehyde, and Amines Condense to Give the Proposed Azadithiolate Cofactor of the Fe-Only Hydrogenases. *J. Am. Chem. Soc.* **2002**, *124* (5), 726-727.

(3) Le Cloirec, A.; Best, S. P.; Borg, S.; Davies, S. C.; Evans, D. J.; Hughes, D. L.; Pickett, C. J. A Di-Iron Dithiolate Possessing Structural Elements of the Carbonyl/Cyanide Sub-Site of the H-Centre of Fe-Only Hydrogenase. *Chem. Commun. (Cambridge, U. K.)* **1999**, (22), 2285-2286.

(4) Lowry, O.; Rosebrough, N.; Farr, A. L.; Randall, R. Protein Measurement with the Folin Phenol Reagent. *J. Biol. Chem.* **1951**, *193* (1), 265-275.

(5) Siritanaratkul, B.; Megarity, C. F.; Roberts, T. G.; Samuels, T. O. M.; Winkler, M.; Warner, J. H.; Happe, T.; Armstrong, F. A. Transfer of Photosynthetic NADP(+)/NADPH Recycling Activity to a Porous Metal Oxide for Highly Specific, Electrochemically-Driven Organic Synthesis. *Chem Sci* **2017**, *8* (6), 4579-4586.

(6) Adamska, A.; Silakov, A.; Lambertz, C.; Rudiger, O.; Happe, T.; Reijerse, E.; Lubitz, W. Identification and Characterization of the "Super-Reduced" State of the H-Cluster in [FeFe] Hydrogenase: A New Building Block for the Catalytic Cycle? *Angew. Chem. Int. Ed. Engl.* **2012**, *51* (46), 11458-11462.

(7) Daido, T.; Akaike, T. Electrochemistry of Cytochrome C: Influence of Coulombic Attraction with Indium Tin Oxide Electrode. *J. Electroanal. Chem.* **1993**, *344* (1), 91-106.

(8) Fourmond, V. Qsoas: A Versatile Software for Data Analysis. *Anal. Chem.* **2016**, *88* (10), 5050-5052.

(9) Stoll, S.; Schweiger, A. Easyspin, a Comprehensive Software Package for Spectral Simulation and Analysis in Epr. *J. Magn. Reson.* **2006**, *178* (1), 42-55.

(10) Rodriguez-Macia, P.; Pawlak, K.; Rudiger, O.; Reijerse, E. J.; Lubitz, W.; Birrell, J. A. Intercluster Redox Coupling Influences Protonation at the H-Cluster in [FeFe] Hydrogenases. *J. Am. Chem. Soc.* **2017**, *139* (42), 15122-15134.

(11) Smith, E. T.; Feinberg, B. A. Redox Properties of Several Bacterial Ferredoxins Using Square Wave Voltammetry. *J. Biol. Chem.* **1990**, *265* (24), 14371-14376.

(12) Maiocco, S. J.; Arcinas, A. J.; Booker, S. J.; Elliott, S. J. Parsing Redox Potentials of Five Ferredoxins Found within Thermotoga Maritima. *Protein Sci.* **2019**, *28* (1), 257-266.

(13) Lampret, O.; Esselborn, J.; Haas, R.; Rutz, A.; Booth, R. L.; Kertess, L.; Wittkamp, F.; Megarity, C. F.; Armstrong, F. A.; Winkler, M.; Happe, T. The Final Steps of [FeFe]-Hydrogenase Maturation. *Proc. Natl. Acad. Sci. U. S. A.* **2019**, *116* (32), 15802-15810.

(14) Megarity, C. F.; Esselborn, J.; Hexter, S. V.; Wittkamp, F.; Apfel, U. P.; Happe, T.; Armstrong, F. A. Electrochemical Investigations of the Mechanism of Assembly of the Active-Site H-Cluster of [FeFe]-Hydrogenases. *J. Am. Chem. Soc.* **2016**, *138* (46), 15227-15233.

(15) Siritanaratkul, B.; Megarity, C. F.; Herold, R. A.; Armstrong, F. A. Interactive Biocatalysis Achieved by Driving Enzyme Cascades inside a Porous Conducting Material. *Commun. Chem.* **2024**, *7* (1), 132.

(16) Alonso-Lomillo, M. A.; Rüdiger, O.; Maroto-Valiente, A.; Velez, M.; Rodríguez-Ramos, I.; Muñoz, F. J.; Fernández, V. M.; De Lacey, A. L. Hydrogenase-Coated Carbon Nanotubes for Efficient H2 Oxidation. *Nano Lett.* **2007**, *7* (6), 1603-1608.

(17) Plumere, N.; Rudiger, O.; Oughli, A. A.; Williams, R.; Vivekananthan, J.; Poller, S.; Schuhmann, W.; Lubitz, W. A Redox Hydrogel Protects Hydrogenase from High-Potential Deactivation and Oxygen Damage. *Nat. Chem.* **2014**, *6* (9), 822-827.

(18) Oughli, A. A.; Conzuelo, F.; Winkler, M.; Happe, T.; Lubitz, W.; Schuhmann, W.; Rudiger, O.; Plumere, N. A Redox Hydrogel Protects the O2 -Sensitive [FeFe]-Hydrogenase from Chlamydomonas Reinhardtii from Oxidative Damage. *Angew. Chem. Int. Ed. Engl.* **2015**, *54* (42), 12329-12333.

(19) Fourmond, V.; Stapf, S.; Li, H.; Buesen, D.; Birrell, J.; Rüdiger, O.; Lubitz, W.; Schuhmann, W.; Plumeré, N.; Léger, C. Mechanism of Protection of Catalysts Supported in Redox Hydrogel Films. *J. Am. Chem. Soc.* **2015**, *137* (16), 5494-5505.
